# Economic evaluation of Community Level Interventions for Pre-eclampsia (CLIP) in South Asian and African countries: a study protocol

**DOI:** 10.1186/s13012-015-0266-5

**Published:** 2015-05-26

**Authors:** Asif R. Khowaja, Craig Mitton, Stirling Bryan, Laura A. Magee, Zulfiqar A. Bhutta, Peter von Dadelszen

**Affiliations:** Department of Obstetrics and Gynaecology; and Child and Family Research Institute, University of British Columbia, Vancouver, Canada; School of Population and Public Health, University of British Columbia, Vancouver, Canada; Centre for Clinical Epidemiology and Evaluation, Vancouver Coastal Health Research Institute, Vancouver, Canada; Division of Women and Child Health, Aga Khan University, Karachi, Pakistan; Centre for Global Child Health, Hospital for Sick Children, University of Toronto, Toronto, Canada

**Keywords:** Economic evaluation, Cost-effectiveness, Community-based interventions, Pre-eclampsia, Low-middle-income countries

## Abstract

**Background:**

Globally, hypertensive disorders of pregnancy, particularly pre-eclampsia and eclampsia, are the leading cause of maternal and neonatal mortality, and impose substantial burdens on the families of pregnant women, their communities, and healthcare systems. The Community Level Interventions for Pre-eclampsia (CLIP) Trial evaluates a package of care applied at both community and primary health centres to reduce maternal and perinatal disabilities and deaths resulting from the failure to identify and manage pre-eclampsia at the community level. Economic evaluation of health interventions can play a pivotal role in priority setting and inform policy decisions for scale-up. At present, there is a paucity of published literature on the methodology of economic evaluation of large, multi-country, community-based interventions in the area of maternal and perinatal health. This study protocol describes the application of methodology for economic evaluation of the CLIP in South Asia and Africa.

**Methods:**

A mixed-design approach i.e. cost-effectiveness analysis (CEA) and qualitative thematic analysis will be used alongside the trial to prospectively evaluate the economic impact of CLIP from a societal perspective. Data on health resource utilization, costs, and pregnancy outcomes will be collected through structured questionnaires embedded into the pregnancy surveillance, cross-sectional survey and budgetary reviews. Qualitative data will be collected through focus groups (FGs) with pregnant women, household male-decision makers, care providers, and district level health decision makers. The incremental cost-effectiveness ratio will be calculated for healthcare system and societal perspectives, taking into account the country-specific model inputs (costs and outcome) from the CLIP Trial. Emerging themes from FGs will inform the design of the model, and help to interpret findings of the CEA.

**Discussion:**

The World Health Organization (WHO) strongly recommends cost-effective interventions as a key aspect of achieving Millennium Development Goal (MDG)-5 (i.e. 75 % reduction in maternal mortality from 1990 levels by 2015). To date, most cost-effectiveness studies in this field have focused specifically on the diagnostic and clinical management of pre-eclampsia, yet rarely on community-based interventions in low-and-middle-income countries (LMICs). This study protocol will be of interest to public health scientists and health economists undertaking community-based trials in the area of maternal and perinatal health, particularly in LMICs.

**Trial registration:**

ClinicalTrials.gov: NCT01911494

## Background

Globally, hypertensive disorders of pregnancy (HDP), particularly pre-eclampsia and eclampsia, are the leading cause of maternal and neonatal mortality and impose substantial burdens on the families of pregnant women, their communities, and healthcare systems [1, 2]. Pre-eclampsia occurs when the pregnant woman has concurrent hypertension and significant proteinuria [3]. In the absence of early identification and timely case management of pre-eclampsia, the trajectory can put women at high risk of life-threatening complications [4]. Each year, it is estimated that HDP complicates 10 million pregnancies, resulting in 76,000 maternal and 500,000 foetal/newborn deaths [5]. Nearly all of these deaths (>99 %) occur in low-and-middle-income countries (LMICs), particularly in South Asia and Sub-Saharan Africa [6].

Previously, the definitive management of HDP has focused on health facility level interventions with antihypertensive [7] and anticonvulsant [8] therapies and timed delivery to reduce risks. However, thousands of women in hard-to-reach areas in resource-constrained LMICs continue to suffer severe disability or lose their lives because of delays in early identification, triage, transport and treatment—clinical processes that could feasibly be managed within a woman’s own community at the level of a primary health centre (PHC) or nearby via admission to a more central referral hospital [9].

### The CLIP Trial

The Community Level Interventions for Pre-eclampsia (CLIP) is an ongoing cluster randomized trial [ClinicalTrials.gov number ID NCT01911494] [10] that introduces evidence-based interventions applied at both community and PHC levels to reduce maternal and perinatal disabilities and deaths resulting from the failure to identify and manage pre-eclampsia at the community level. Specifically, the CLIP intervention consists of:

I. *Community engagement* including women from the communities, dyadic household decision-makers (husbands, fathers-in-law) and community leaders about: pre-eclampsia, its origins, symptoms, signs and potential consequences, pre-permissions for maternal transport and fundraising activities for transport and treatment costs;
II. *Provision of HDP-oriented antenatal care through household visits* by community healthcare providers (cHCPs) who carry a mobile health application for identifying women at risk of pre-eclampsia [Pre-eclampsia Integrated Estimate of Risk (PIERS) [11] on the Move (POM) [12] app];
III. *Use of the CLIP package for women with a CLIP ‘trigger’* (i.e. oral antihypertensive therapy or intramuscular (i.m.) magnesium sulphate (MgSO_4_) when indicated, and appropriate referral to a comprehensive emergency obstetric care (CEMOC) facility as needed).

The cHCPs assess pregnant women with a target frequency of every 4 weeks at a minimum. These visits can occur in the home or PHC, which are both considered part of the community for the purpose of the CLIP Trial. The cHCPs are trained to enquire about the woman’s symptoms (using country-specific pictograms), take blood pressure and check urine for protein using a dipstick on the first visit or on any subsequent visits if the systolic blood pressure is ≥140 mmHg. This helps to inform diagnosis of and risk assessment for pre-eclampsia. The control group (without intervention) continues with routine pregnancy care related to antenatal visits, referral to a health facility and initiation of therapy.

### Cost and cost-effectiveness of interventions for pre-eclampsia/eclampsia

Pre-eclampsia imposes very high financial burdens on the families of the affected women and on the healthcare system in LMICs [2, 13]. Economic studies conducted in the United Kingdom (UK) report that pre-eclampsia/eclampsia is one of the most common reasons for antenatal admission to hospital (20 %) and of obstetric admissions to intensive care units (25 %) [14]. Other studies from the US report that hospitalization costs for the management of pre-eclampsia and associated complications were US$11,208 per woman on average [15, 16]. Studies from LMICs report death or surviving serious illness of a mother to result in lower household income [17] and to raise the risk of death for children aged <10 years [18].

There are few cost-effectiveness studies related to pre-eclampsia/eclampsia. Existing studies have focused on diagnostic and clinical interventions in well-resourced settings and not on other issues at community or population levels. For example, a recent study from Israel evaluated the economic benefit of first-trimester screening of multiple markers compared with no screening. This study found a cost per quality-adjusted life year (QALY) less than US$10,000 for screening, given the prevalence of pre-eclampsia at 3 % [19].

Another economic study in the context of the UK’s National Health Service reported that the protein-creatinine ratio (Pr:Cr) alone, when compared with automated reagent-strip reading device followed by Pr:Cr and/or 24-h measurement of proteinuria, was found less costly and gained the most QALYs [20].

A large multi-country trial on prophylactic use of MgSO_4_ in women with pre-eclampsia reported the incremental cost of preventing one case of eclampsia as US$21,202 in high-income, US$2473 in middle-income and US$456 in low-income countries [21]. Another study from the UK found that the prophylactic administration of MgSO_4_ to all women with pre-eclampsia had an incremental cost of US$9994 for each additional seizure prevented [22].

Other interventions in the context of the UK, such as treatment with aspirin compared with no aspirin, were found to be cost saving (i.e. £7852) and resulted in 0.52 additional QALYs per pregnancy in women at risk of pre-eclampsia [23]. The labour induction for immediate birth versus clinical management strategy was found to have an incremental cost-effective ratio (ICER) of £2900 per QALY gained in women diagnosed with mild or moderate pre-eclampsia [24].

Cost-effectiveness studies have also evaluated other interventions that can be used to improve maternal [25] and neonatal health [26] in LMICs. However, their relevance to pre-eclampsia/eclampsia interventions is limited given the restricted analysis of individual interventions on surrogate health outcomes and the variability of settings.

In relation to the proposed work, our literature review found very limited information for cost-effective interventions for pre-eclampsia/eclampsia in the context of LMICs, which is where most of the disease burden and associated mortality occur. Thus, it is critical to conduct an economic evaluation alongside of the CLIP Trial, to inform decision makers not only of clinical outcomes but the cost required to obtain those outcomes.

### Rationale for conducting economic evaluation of CLIP

Adam et al. [27] conducted a cost-effectiveness analysis of WHO-recommended strategies for maternal and neonatal health and demonstrated the benefits of comprehensive community-based antenatal, intrapartum and postnatal interventions for reducing maternal and neonatal mortality in sub-Saharan Africa and Southeast Asia. They highlighted that packages of maternal and newborn interventions can be more cost-effective than singular interventions.

In our context, the CLIP Trial combines a package of otherwise singular evidence-based interventions (blood pressure monitoring [15], urine dipstick testing [28], MgSO_4_ [21], methyldopa [29], mobile health (mHealth) technology [12, 30], antenatal visits by cHCPs [18], community engagement [31], timely referral and triage at a health facility [6]). This combined package of care must be evaluated to determine if it is a cost-effective intervention in reducing maternal and perinatal mortality.

It is well recognized that economic studies embedded within clinical trials have high internal validity and timeliness [32]. In this context, the International Society for Pharmacoeconomics and Outcome Research (ISPOR) recommended collecting trial outcome data, health resources used and health state utilities directly from the study participants recruited in the trial [33].

The CLIP Trial is being conducted across four countries, and so assessment of the economic impact (costs and benefits) alongside the trial (i.e. concurrent with it) will be integral to building a robust cost-effectiveness model to supplement the trial outcomes. This is critical because the CLIP Trial introduces new costs in health service delivery (e.g. mHealth and task shifting to cHCPs), which will have budgetary implications for health systems in the selected CLIP countries. Post-trial program scale-up of CLIP interventions must be informed through both the impact of the package of intervention *and* the cost of achieving any incremental benefits in the context of selected South Asian and African countries.

The CLIP Trial’s collaborating partners and Ministries of Health (MoH) in the respective countries have a clear desire to ascertain the long-term implications of the CLIP package of care. These stakeholders unanimously endorsed conducting an economic appraisal of CLIP to further inform policy decisions around resource allocation and program scale-up, thereby serving as models for other LMICs.

### Study hypothesis

The CLIP package combined with routine pregnancy care, compared to routine pregnancy care alone, will result in a favourable (i.e. low) ICER in reducing maternal and perinatal mortality and major morbidities.

### Study objectives

The *primary objectives* are to:

- Determine the costs and benefits of the CLIP package of care in order to design the cost-effectiveness model;
- Estimate the ICER of the CLIP plus routine pregnancy care, compared with routine pregnancy care alone, in reducing maternal and perinatal mortality and major morbidities;
- Assess health system budget impact, when switching from routine pregnancy care to CLIP plus routine pregnancy care.

The *secondary objectives* are to:

- Qualitatively identify the cost drivers (resources needed) during trial implementation to inform design of the model;
- Explore implementation challenges and perceived cost-benefits of CLIP plus routine pregnancy;
- Inform health decision/policy makers about the findings of cost-effectiveness of the CLIP package of care and budgetary implications in the selected countries.

## Study methods

### Research design

We propose to use CEA in conjunction with qualitative analysis alongside the trial to prospectively evaluate the economic impact of CLIP. Qualitative research has been used effectively in other cluster RCTs to assess the implementation variations and local context of the intervention [34]. We will use a mixed-method approach because the CLIP Trial is being conducted in four countries that have different health delivery systems, health financing, resource allocation interests, diversity of community beliefs about pre-eclampsia/eclampsia, care-seeking behaviours and treatment preferences. The mixed-method approach (CEA and qualitative) will inform the design of modelling and support interpretation of economic analysis for decision makers who are considering evidence of economic value along with the effectiveness of CLIP.

### Research plan

**Fig. 1:**
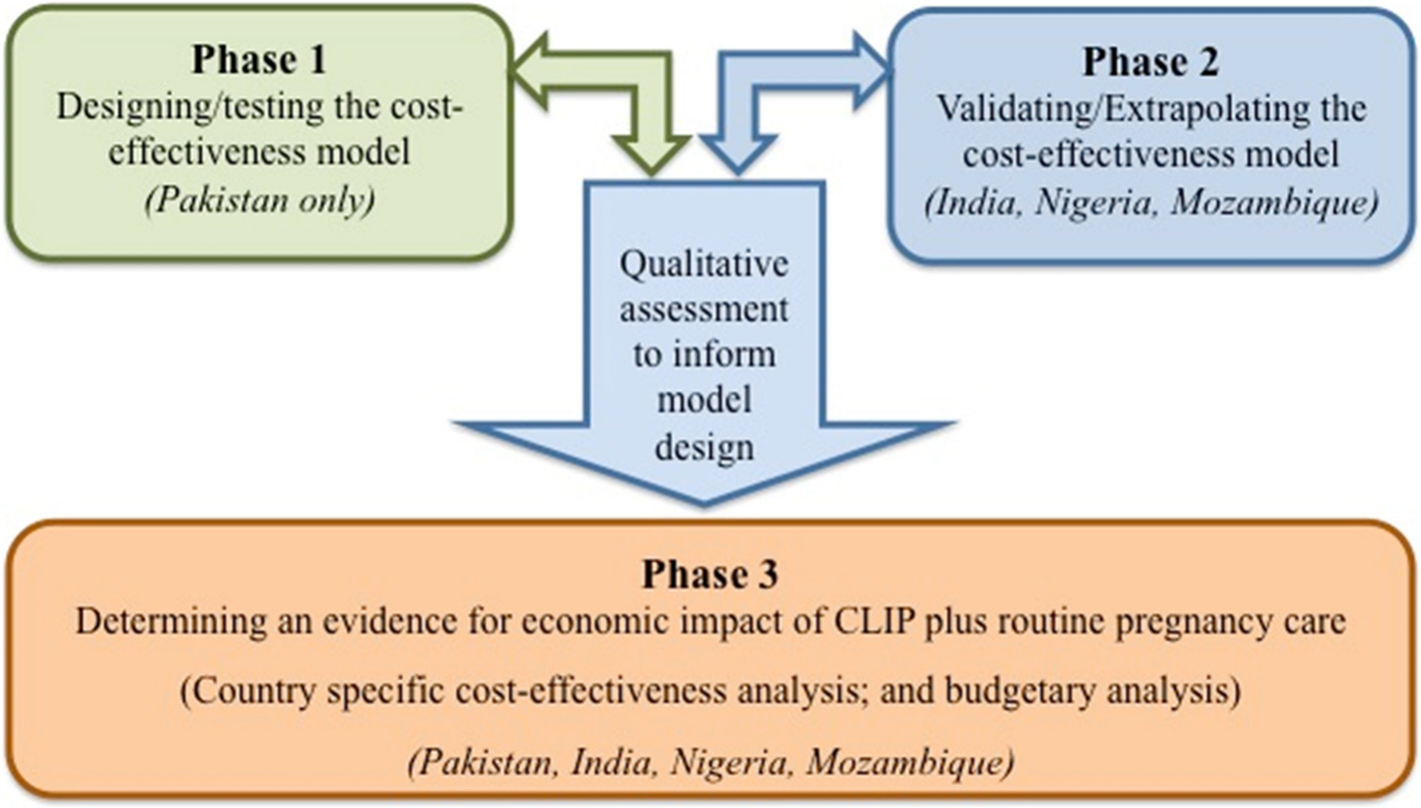
Research plan for economic evaluation of the CLIP

The research will be conducted in three inter-linked phases over a 2-year period. (Please refer to Fig. 1).

*Phase 1* will contribute to objective 1a using cost estimation and outcome monitoring methods to design a cost-effectiveness model for CLIP. Qualitative assessments will address objectives 2a and 2b to understand contextual aspects of the costs, challenges during trial implementation and benefits from community perspectives to support interpretation of the economic analysis. Phase 1 activities will be undertaken in Pakistan, one of the four countries engaged in the CLIP Trial.

In *Phase 2*, based on the learning in Phase 1, we will use similar, but contextually modified, methods guided by within-country data collection to validate/extrapolate the cost-effectiveness model in the other three sites (i.e. India, Nigeria and Mozambique).

*Phase 3* will address Objectives 1b and 1c, once the final trial outcomes are analysed and available to run the cost-effectiveness analysis for all sites. In addition, it will address Objective 2c to inform health decision/policy makers about cost-effectiveness and budgetary implications.

### Target population

Because pre-eclampsia is a pregnancy-related illness, this study will target pregnant women aged 15–49 years (except Mozambique where the eligibility age is 12–49 years), those recruited in the primary CLIP Trial in both intervention and control clusters.

### Settings and duration

The sites for the CLIP Trial include local government areas of Ogun State, Nigeria; the Provinces of Gaza and Maputo in Mozambique; the districts of Matiari and Hyderabad in the Province of Sindh, Pakistan; and the Belgaum and Bagalkot districts in the State of Karnataka, India. The economic evaluation of CLIP will be a critical step to guide policy decisions for post-trial program scale-up in all of these selected countries.

The total duration of the proposed country-specific cost-effectiveness analysis will be 2 years (Sept 2015–Aug 2017) (please refer to Table 1). Phase 1 will commence in September 2015 and will include activities for cost calculation and qualitative assessments at one site. The data from phase 1 will be analysed by April 2016. Phase 2 will commence in May 2016 at the remaining three CLIP sites and shall be completed by December 2016. Finally, the country-specific CEA will be completed by June 2017, followed by report writing and result dissemination in August 2017.

**Table 1:** Project milestones (GANTT chart) for economic evaluation of the CLIP

2015	2016			2017	
1–4 (Sep–Dec)	5–8 (Jan–Apr)	9–16 (May–Dec)		17–22 (Jan–June)	23–24 (Jul–Aug)
*Phase I*		*Phase II*		*Phase III*	
Designing/Testing the costing model (Pakistan only)		Validation/Extrapolation of the costing model (India, Nigeria, and Mozambique)		Determining an evidence for economic impact of the CLIP (All four sites)	
Field level planning; and conducting qualitative focus groups (FGs) and data analysis	Collecting costing data and data analysis	Conducting FGs; and collecting cost data	Data analysis and extrapolation of model	Generating country-specific cost-effectiveness models	Writing report; and conducting final result dissemination seminar
				-Calculating incremental cost per combined maternal/perinatal outcomes averted	
		-Baseline survey for cost estimation			
-FGs with community					
-FGs with health providers -FGD with Policy makers community and health workers					
			-Model validation		
		-Collecting price list for health services			
		-Review of district level cHCP budget			
				-Calculating incremental cost per DALY gained.	
				Country-specific budgetary analysis	
		-FGs with community			
	-Baseline survey for cost estimation				
	-Collecting price list for health services				
	-Review of district level cHCP budget				
	-Designing/Testing the model				
		-FGs with health providers			
		-FGD with Policy makers			

### Study perspective

The CEA will be based on a societal perspective, accounting for both costs to healthcare system and cost to families of the pregnant women.

### Designing the model: description of cost variables

The standardized ingredient approach [35], which involves gathering sufficient information about the quantities and unit cost of physical inputs needed in the intervention and control groups, will be used to calculate costs. This will include cost to the healthcare system and cost to the family.

#### Cost to the healthcare system

The cost to the health system will comprise the cost of the CLIP Trial interventions, including mHealth technology and infrastructure, blood pressure devices, urine dipsticks, community engagement sessions, training and time of healthcare providers at the community and health facility levels. Additional costs will include:

- Cost of follow-up household visits and time spent on blood pressure monitoring/urine dipstick by cHCPs in each of the selected sites, such as Community Health Extension Workers (CHEW) in Nigeria, Agente Polivalente Elementar (community health agents) in Mozambique, Lady Health Workers (LHW) in Pakistan and Accredited Social Health Activists (ASHA) in India;
- Cost of cHCPs’ additional time and transport costs when accompanying any identified HDP woman to a referral health facility;
- Health system costs such as managing triage for obstetric emergencies, in-patient/outpatient services for obstetric emergencies, as well as diagnostic tests and drugs.

#### Cost to the family

All relevant out-of-pocket (OOP) expenses for ambulance, hospitalization (physician fees, bed charges, nursing services), drugs and diagnostic workup related to care from the referral health facility would be included. Also, OOP cost for informal care (i.e. care sought from traditional healers) will be captured, as well as the cost of lost productivity resulting from morbidity or mortality of patients with or without paid jobs, and any lost wages of their caregivers.

#### Cost to society

The total societal costs (i.e. combining of costs to the healthcare system and cost to the family) will be calculated by summing across all cost categories.

### Comparators

This study will compare the costs and pregnancy outcomes ascertained from the intervention i.e. CLIP plus routine pregnancy care; compared with control group i.e. routine pregnancy care alone. In a primary CLIP cluster-randomized trial, the interventions are being evaluated at the population level. Therefore, people in the comparator group will continue to receive routine pregnancy care related to antenatal visits, referral to a health facility and initiation of therapy.

### Health resource utilization and costs: data collection methods

The information about resources utilized and unit costs will be collected from primary and secondary data sources in the intervention and control groups. A consistent approach will be followed to collect these data in the intervention and control clusters, except for POM utilization, which only occurs in the intervention clusters (see Table 2).

**Table 2:** Methods for collecting resource utilization and cost information

Type of data	Intervention group	Control group
Health resource utilization	Health resource utilization questionnaire integrated with CLIP Trial quarterly surveillance tools for intervention and control groups:	
	Form 1: Pregnancy Registration	
	Form 2: Regular community surveillance	
	Form 3: Health facility utilization	
	PIERS on the move (POM) data	N/A
Unit costs	Cross-sectional household survey for family’s out-of-pocket expenses	
	Review of price listing for diagnostic and clinical services offered at health facilities	
	Review of district level program budget (costing for cHCP salaries)	
	Review of site-specific CLIP Trial budget (costing for intervention package)	N/A

#### Health resource utilization data

Structured health resource utilization questionnaires are embedded into CLIP Trial surveillance forms and will be administered to all pregnant women recruited in intervention and control clusters. These questionnaires have been translated into study site languages (Yoruba in Nigeria, Portuguese in Mozambique, Sindhi/Urdu in Pakistan and Kannada in India) and are as follows:

- *Form 1: Pregnancy registration:* Project research staff will complete this form only once for every pregnancy identified during the trial period in the intervention and control groups. The key variables include health resource utilization, such as frequency of hospital visits, type of health facility (public or private), level of health facility (primary, secondary, or tertiary), level of care (in-patient or out-patient), length of stay, diagnostic tests and clinical interventions since conception to the time of pregnancy registration. Also, information will be collected on mode of transport, number of accompanying family members and days of missed wages.
- *Form 2: Regular community surveillance*: Project staff will complete this form once every 3 months until delivery and once post-delivery capturing data for the 42 days after childbirth, for each pregnant woman recruited in the intervention and control groups. The key variables include health resource utilization, such as frequency of hospital visits, type of health facility (public or private), level of health facility (primary, secondary, or tertiary), level of care (in-patient or out-patient), length of stay, diagnostic tests and clinical interventions for pregnant women and newborn. Also, information will be collected on mode of transport used, number of accompanying family members and days of missed wages.
- *Form 3: Health facility utilization*: This is based on patient hospital admission chart review for women recruited in the CLIP Trial and will be completed by project research staff during their monthly visits at all referral health facilities in the catchments of intervention and control groups. The key variables include diagnostic and clinical services utilized by pregnant women and/or newborns at health facilities.

#### The POM data

The information about resource utilization as a result of CLIP interventions will be captured from the POM data set. Based on clinical triggers, CLIP interventions are classified into *five main categories*: 1) treat with MgSO_4_ and transport to hospital; 2) urgent transport (within 4 h) to hospital only; 3) non-urgent transport (within 24 h) to hospital only; 4) treat with MgSO_4_ and methyldopa and transport to hospital; and 5) continue with routine antenatal care. POM data is maintained electronically for all the women recruited in the intervention clusters.

#### Cross-sectional household survey

The survey will be conducted in a sample of women from intervention and control clusters to determine unit costs for out-of-pocket expenses to the family associated with obstetric emergencies including HDP. The value of the lost wages will be estimated by using a mean wage rate to missed work time, obtained from country-specific standards. Assuming an incidence rate of pre-eclampsia at 8 %, 95 % confidence interval and design effect of 1.0 for a simple random sample, the required sample size for baseline survey at each site is presented in Table 3.

**Table 3:** Site-specific population and desired number of women for survey

Variables	Pakistan	India	Nigeria	Mozambique	Total
Total population per cluster	32,000	27,000	70,000	25,000	*154,000*
Number of intervention and control clusters	20	12	10	12	*54*
Annual birth rate (/1000/year)	14	22	16	40	*92*
Total number births during trial period	19,800	19,200	26,880	24,400	*90,280*
Estimated number of women with pre-eclampsia during trial period	1584	1536	2150	1952	*7220*
*Number of women required for survey*	*113*	*113*	*136*	*126*	*488*

#### Review of price listing for maternal and newborn health services

In order to obtain the unit cost of hospitalizations (e.g. bed charges, nursing services), drugs and diagnostics, the price lists will be obtained from all public and private health facilities where women with HDP will be referred in the catchments of intervention and control clusters. The weighted average will be calculated for estimating unit costs for similar types of services available at private and public health facilities, with respect to the number of women utilizing such services for pregnancy care, delivery and newborn care.

#### Reviewing district level cHCP program budget

The salaries of cHCPs who are currently involved in the CLIP Trial will be determined through review of the district level program budget. Where resources are shared with other preventive programs, we will use simultaneous allocation methods for determining the unit cost of applicable services. In addition, the transport expenses will be calculated for the extra visits of cHCPs in the intervention clusters to be able to determine the cost of task shifting.

#### Review of site-specific CLIP Trial budget

The unit cost estimates for the CLIP Trial intervention package include the cost of a blood pressure device, urine dipstick, oxygen saturation prop, cost of community engagement sessions, and cost of training doctors, nurses, midwives and community health workers and will be determined from the trial budget for each site in the CLIP Trial. These cost estimates will be verified from the central trial office (PRE-EMPT, UBC).

### Designing the model: qualitative data collection methods

Focus groups (FGs) are a commonly used method of data collection in qualitative research to gather group opinions [36]. Specifically, the FGs in this study are aimed to better understand the contextual variations of intervention delivery, resources used for costing work and perceived benefits from a community perspective. Besides healthcare providers and care receivers in a community, studies have reported that women in LMICs are situated in cultural contexts, where men in their lives are traditionally the decision makers surrounding women’s health issues [37]. As inclusively as possible, the community perspectives will be obtained from groups of:

- Pregnant women identified as at risk due to a HDP;
- Male decision makers (husbands/fathers-in-law) of pregnant women identified as at risk of a HDP;
- Community healthcare providers;
- Medical doctors at referral health facilities;
- District-level health decision/policy makers.

Guided by relevant literature [37, 38], and investigators’ experiences of CLIP Trial feasibility work in the CLIP countries, semi-structured interview guides have been developed following a priori themes:

- Theme I: Cost drivers and health resource utilization as a result of the CLIP package.
- Theme II: Perceived benefits of the CLIP package of care and task shifting to cHCPs.
- Theme III: Implementation challenges for the CLIP package of care.
- Theme IV: Strategies for knowledge translation of CLIP to the wider community.
- Theme V: Strategies for health policy advocacy and program scale-up of CLIP.

The FG guides will be translated into local languages and pilot tested in randomly selected intervention clusters before data collection. Digital voice recorders and written notes will be used to record the participants’ responses during all FGs. The FG data will be transcribed into the local language, followed by translation into English. All the translations will be confirmed by researchers with back-translation of randomly selected data segments for quality control.

### Study eligibility (inclusion and exclusion criteria)

Pregnant women aged 15–49 years (except Mozambique where the eligibility age is 12–49 years) recruited in the CLIP Trial in both intervention and control clusters will be eligible to take part in the economic data collection (i.e. Form 1, Form 2 and Form 3) for health resource utilization. For qualitative assessments, eligible participants include only women in the CLIP intervention group who were identified as at risk of a HDP. Likewise, male decision makers of pregnant women (identified as at risk of a HDP) and those willing to participate in the session will be eligible. The cHCP handling the CLIP Trial package of intervention, the medical doctors at the referral health facilities and district health decision makers in the catchments of intervention and control groups and those willing to participate in 45- to 60-min session will be eligible to participate. Participants who will be excluded are those who are not recruited in the primary CLIP Trial and/or refused to take part in the economic data collection procedures. The eligible participants for qualitative assessments will be approached by project research staff during home and health facility visits for CLIP Trial surveillance and will be invited to participate in FGs.

### Sample size

The sample size required to demonstrate the effectiveness of the primary CLIP Trial will be sufficient as it is powered to detect 30 % effect size. We estimate a total of 90,000 pregnant women will be registered in the CLIP Trial across four sites; and those who consent to surveillance (Form 1, 2, and 3) will be included in our economic analysis.

Each FG will include 6–9 participants. We anticipate a total of 40 FGs inclusive of all groups; however, the final number of FGs will be determined by data saturation (see Table 4).

**Table 4:** Number and distribution of FGs across CLIP countries

CLIP country	Number of focus groups (anticipated)					Total
	Pregnant women with HDP	Male decision makers	Community healthcare providers	Doctors at health facilities	District health decision makers	
Pakistan	2	2	2	2	2	10
India	2	2	2	2	2	10
Nigeria	2	2	2	2	2	10
Mozambique	2	2	2	2	2	10
*Total*	*8*	*8*	*8*	*8*	*8*	*40*

### Outcome variables (effectiveness): methods of collecting outcomes

The CLIP Trial primary outcome is the reduction in combined maternal and/or perinatal adverse outcomes between the intervention and control groups (see Table 5). The project research staff will assess the trial outcomes every 3 months during community surveillance visits at the households for all women recruited in the intervention and controls groups.

**Table 5:** Definitions of CLIP Trial outcomes

	Definitions
Maternal outcomes	
Mortality	Defined as the number of deaths during pregnancy or within 42 days of pregnancy (or last contact day if contact not maintained to 42 days)/1000 identified pregnancies), termed maternal death rate.
Morbidities	Defined as the number of women with one or more life-threatening complications of pregnancy during pregnancy or within 42 days of pregnancy/1000 identified pregnancies.
	Serious end-organ complications of pre-eclampsia:
	Eclampsia: occurrence of generalized convulsions during pregnancy, labour or within 42 days of delivery in the absence of epilepsy or another condition predisposing to convulsions
	Stroke: hemiparesis and/or blindness developed during pregnancy or in the 42 days postpartum lasting greater than 48 h
	Coma: prolonged unconsciousness ≥12 h
	Antepartum haemorrhage: vaginal bleeding ≥15 mL with or without pain before the onset of labour
	Disseminated intravascular coagulation (DIC): abnormal bleeding from mucosa (mouth and/or ears)
	Other major causes of maternal mortality:
	Obstetric sepsis: In the community, defined as fever and one of: abdominal/uterine tenderness, foul smelling vaginal discharge/lochia, productive cough and shortness of breath, dysuria or flank pain, headache and neck stiffness. In the facility, defined as presence of fever (>38 °C), a confirmed or suspected infection (e.g. chorioamnionitis, septic abortion, endometritis, pneumonia) and at least one of the following: heart rate >90/min, respiratory rate >20/min, leukopoenia (total leukocyte count [TLC] <4 × 10^9^/L) or leukocytosis (TLC >12 × 10^9^/L)
	Vesicovaginal or rectovaginal fistula: continuous loss of urine and/or faeces after delivery
	Life-saving interventions:
	Cardiopulmonary resuscitation: a set of emergency procedures including chest compressions and lung ventilation applied in cardiac arrest victims
	Dialysis: haemodialysis and/or peritoneal dialysis
	Mechanical ventilation (other than for Caesarean section): intubation and ventilation not related to anaesthesia
	Blood transfusion: ≥1 unit
	Interventions for major postpartum haemorrhage: brace sutures, external and internal uterine compression, anti-shock garment use, internal iliac artery ligation and/or hysterectomy with or without transfusion
Perinatal outcomes	
Mortality	Defined as stillbirth [≥20^+0^ and/or ≥500 g], early neonatal mortality [days 0–7 of postnatal life] and late neonatal mortality [days 8–28 of postnatal life]/1000 identified pregnancies]
Morbidity	Defined as non-lethal events of seizure and coma during days 0–28 of postnatal life/1000 identified pregnancies). The following are the primary neonatal morbidities:
	Feeding difficulty
	Breathing difficulty
	Seizure
	Lethargy
	Coma
	Fever
	Hypothermia
	Umbilical cord infection
	Skin infection
	Bleeding
	Jaundice
	Vomiting/Diarrhoea

### Data analysis

**Fig. 2:**
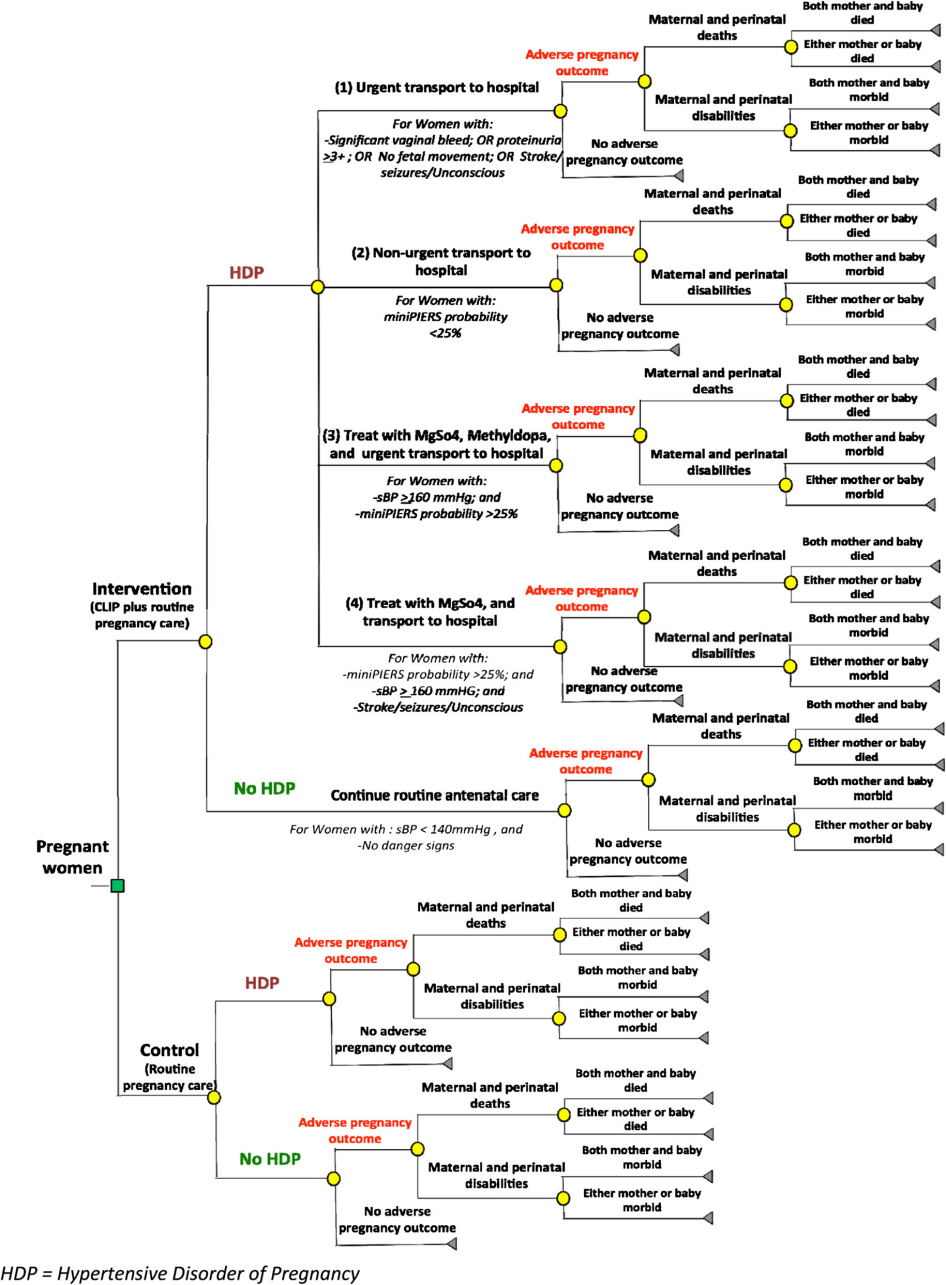
Decision analytic tree model for economic evaluation of CLIP. *HDP*, hypertensive disorder of pregnancy

Because outcomes of pregnancy can be assessed over a short span (i.e. 40–42 weeks of time horizon), the decision analytic tree model [39] can be used for comparative analysis of costs and effectiveness between two alternatives. Previously conducted cost-effectiveness studies for pre-eclampsia [19, 22] have mainly used a decision analytic tree model. Our primary analysis for this study will be model based, guided by previous work in high-income countries as no LMIC modelling in pre-eclampsia has been done. We will use parameter estimates for costs and effectiveness coming from the CLIP Trial (see Fig. 2).

The unit costs will be multiplied by identified health resource utilization to calculate the total cost per pregnancy, including both pregnant woman and newborns. The total cost will be calculated as the sum of the health resource utilization cost, cost of implementing the CLIP package of care, cost of routine pregnancy care and societal costs. The annual equivalent costs in local currency of selected CLIP countries (PKR—Pakistani Rupee; INR—Indian Rupee, NGN—Nigerian Naira and MZN—Mozambican Metical) will be converted in US dollar exchange rate as of 2015. The pregnancy outcomes (i.e. health of mother and baby) will be modelled as the effectiveness of the CLIP interventions. This will include no-adverse outcomes (healthy mum and newborn at the time of delivery) and adverse outcomes (death and/or disability of mother and baby) observed in the intervention and control groups.

In addition, costs and pregnancy outcomes will be modelled over the lifetime horizon to estimate the long-term impact of CLIP. We will calculate disability-adjusted life years (DALYs)^1^ averted through subsequent modelling to yield estimates of the years of life lost due to disability [40] taking into account the epidemiological rates and health state valuations on burden of disease for African and South Asian regions [41, 42]. Both costs and outcomes will be discounted at 3 % per year, the widely cited discount estimate for economic studies in the context of LMICs [26, 43].

Using the data and parameter estimates specific to each CLIP country, the ICERs will be calculated first from a healthcare system perspective and then from a societal perspective. The country-specific ICER will be calculated as follows:

(i) Incremental cost per adverse pregnancy outcome
(ii) Incremental cost per DALY gained

ICERs for the system perspective as the reference case will be of interest to country-specific health policy makers for resource allocation decisions, when switching from routine pregnancy care to CLIP plus routine pregnancy care, should CLIP be found effective. Critically, however, the ICER from a societal perspective will facilitate discourse on the full opportunity cost in the context of the selected CLIP country. In accord with the recommendation of the Commission for Macroeconomics and Health [44], we will compare the country-specific ICER with the per capita value for the gross national income of each of the four selected CLIP countries for the year 2015.

Given the uncertainties involved in CEA, we will use probabilistic sensitivity analysis to produce cost-effectiveness plots [45]. The confidence region surrounding the cost-effectiveness ratio will be estimated using appropriate statistical methods, including bootstrap and Monte Carlo simulations. Life tables based on data from the World Health Organization’s Southeast Asia and African regions [46] or the West level-26 model [47] will also be used in a sensitivity analysis. Children in LMICs bear a disproportionately large share of the total disease burden, because of the cause structure of the disease burden by age could influence overall distribution of DALYs [48]. As reported on previous cost-effectiveness studies in LMIC, no-age-weighting in the reference case was used on sensitivity analysis [43]. Country-specific health system budget impact analysis will be conducted to facilitate policy decisions for resource allocation, when switching from routine pregnancy care to CLIP plus routine care, should the intervention be found effective.

Qualitative data will be analysed using QSR NVivo v10 software, and responses will be coded to form similar categories. These will be refined through thematic analysis. Data will be interpreted through close communication between local researchers and international team to ensure accuracy.

### Ethical considerations

This study will utilize the existing CLIP Trial infrastructure in the four countries and follow the human ethics protocol for the randomized control trial. There is no physical harm and risk to participants in this economic study. The Institutional Review Board (IRB) approval from UBC has been obtained [ETHICS # H12-00132] for the economic evaluation of the CLIP. In addition, the country-specific formal letters of support and permission for data access have been received from government health authorities in all four CLIP countries. Study questionnaire and data forms will be kept in a secured place accessible only to study staff. No personal identifiers will be used in any reports or publications. Study procedures and benefits will be explained to the study participants, and written consent will be obtained for the baseline cost estimation survey and for qualitative FGs in this study.

### Trial status

The CLIP Trial has been registered at ClinicalTrials.gov (NCT01911494). It is funded by the Bill & Melinda Gates Foundation. The definitive phase of the CLIP Trial is currently recruiting at all four sites and shall continue until December 2016. The recruitment began in India on 1 November 2014, in Pakistan on 19 January 2015, in Mozambique on 1 March 2015 and in Nigeria on 15 March 2015.

## Discussion

**Fig. 3:**
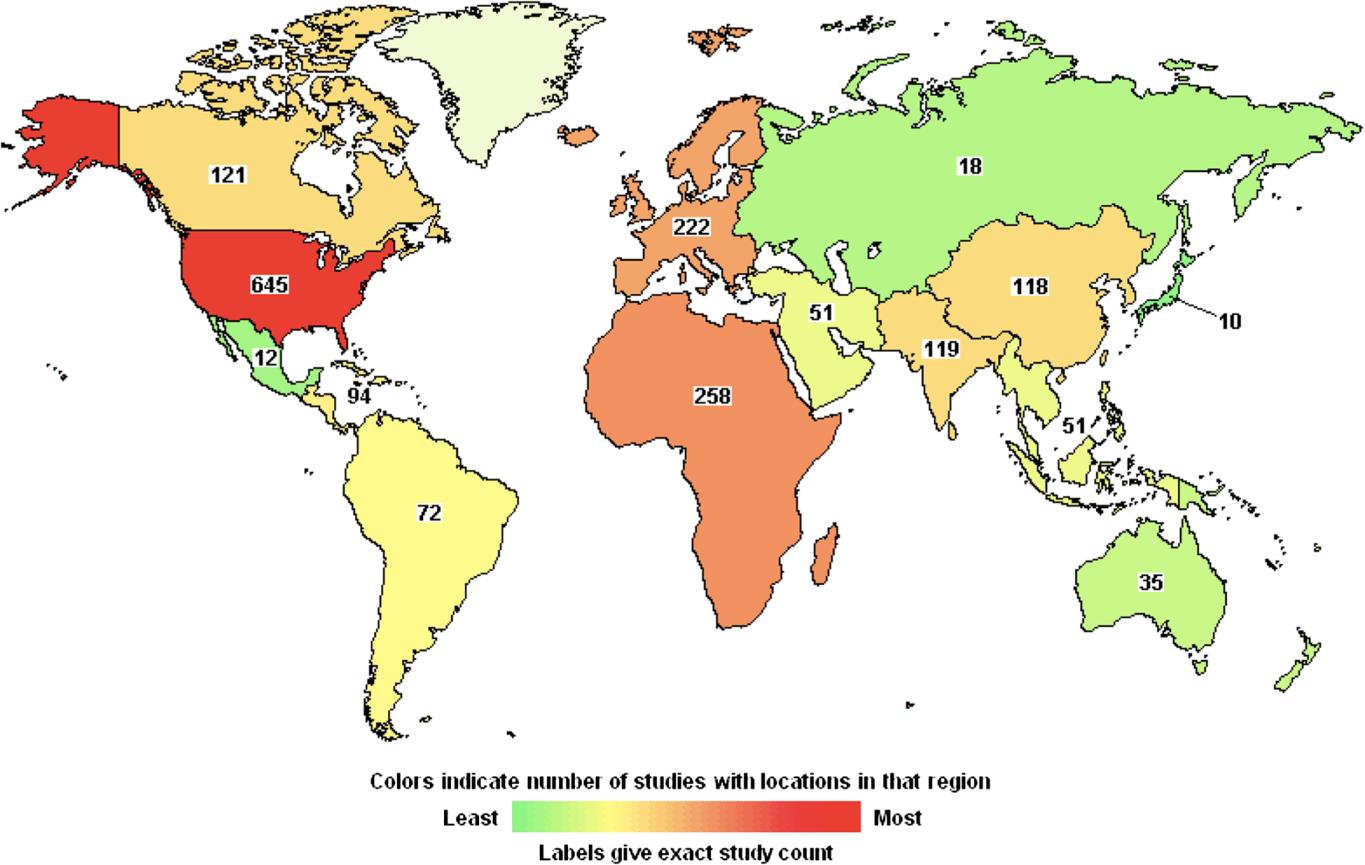
Map of interventional studies on the topic of maternal health—those registered with ClinicalTrials.gov

Economic evaluation of innovative health interventions can play a pivotal role in priority setting and can inform healthcare decision makers with evidence relevant to resource allocation [49]. The World Health Organization (WHO) strongly recommends cost-effective interventions as a key aspect of achieving Millennium Development Goal (MDG)-5 (i.e. 75 % reduction in maternal mortality from 1990 levels by 2015) [50]. Our search for intervention studies registered on ClinicalTrials.gov revealed 1588 studies that mentioned “maternal health” on the submitted protocols [51]. Of these, 36 % were reported from Africa and South Asia, which is where most of the maternal mortality burden occurs [1] (see Fig. 3). Only 28 out of 1588 (i.e. ~2 %) included “cost-effectiveness analysis” on the submitted protocols.

Given this, it seems that few recent maternal health intervention studies have looked at how cost-effectiveness these interventions may be to scale up. This is contrary to the recommendations of WHO for maternal health interventions and indicates a large knowledge gap for policy decisions. Consequently, the CLIP Trial is one of the first large-scale research trials to address this important question in the real-world context in LMICs, and thus provides a unique and timely opportunity for economic evaluation of a well-designed community-based trial that directly aligns with MDG-5.

### Possible study limitations

There is a possibility of recall bias for the cost to family obtained on the baseline survey; however, we will limit the recall length to the most recent hospitalization. In addition, some non-financial factors, such as patient and provider preferences and altruism, could possibly influence families’ healthcare utilization. The methodological consistency in collecting costs and outcomes in both intervention and control groups, supplemented by the qualitative data for designing the cost-effectiveness model for each site, will increase the internal study validity.

### Knowledge translation

Results of the economic impact analyses will be communicated to community stakeholders, providers and policy makers through dissemination workshops organized in each country site after CEA results have been completed. Policy briefs [52] will also be drafted to highlight key findings, and they will be distributed among healthcare providers and district health authorities in other provinces in each country. The progress of the study, findings from interim analysis and final CEA results, will be presented at relevant national/international conferences. Manuscripts will be submitted for publication in peer-reviewed open access journals.

### Potential impact

This study will definitively determine the costs incurred by the CLIP package of care and the number of maternal/perinatal deaths and major morbidities averted per US dollar. In the long run, this study will supplement the scientific evidence around the effectiveness of the intervention and so facilitate policies which best mobilize local technology and employ human resources for maximizing healthy pregnancy outcomes in the selected LMIC countries. The qualitative findings will help to interpret the cost benefit findings of the CLIP Trial, to identify challenges during trial implementation and to strategize knowledge translation for strengthening policy advocacy for post-trial programmatic scale-up and sustained implementation within existing maternal health policies in the selected CLIP countries. Furthermore, we will have robust data to propagate similar models for other non-CLIP LMICs in the developing world.

## Abbreviations

ASHA: accredited social health activists
ANM: auxiliary nurse midwives
APE: agente polivalente elementar
CLIP: community level interventions for pre-eclampsia
CRRH: Centre for Research in Reproductive Health
CISM: The Manhiça Health Research Centre
CHEW: community health extension workers
DALYs: disability-adjusted life years
FGD: focus group discussions
ICER: incremental cost-effective ratio
IRB: institutional review board
ISPOR: International Society for Pharmacoeconomics and Outcome Research
LMIC: low- and middle-income countries
PHC: primary health centre
CEMOC: comprehensive emergency obstetric care
LHW: lady health worker
MoH: Ministry of Health
NPT: normalization process theory
OOP: out-of-pocket
PIERS: pre-eclampsia integrated estimate of risk
QALYs: quality-adjusted life years
RCT: randomized control trials
SPA: service provision assessment

## Footnotes

1 DALYs are defined as a measure of overall disease burden, expressed as the number of years lost due to ill health, disability or early death.
